# Cellular receptors for enterovirus A71

**DOI:** 10.1186/s12929-020-0615-9

**Published:** 2020-01-10

**Authors:** Kyousuke Kobayashi, Satoshi Koike

**Affiliations:** Neurovirology Project, Tokyo Metropolitan Institute of Medical Science, 2-1-6 Kamikitazawa, Setagaya-ku, Tokyo, 156-8506 Japan

**Keywords:** Enterovirus 71, Hand, foot, and mouth disease, Neurological disease, Viral receptor, attachment, Uncoating, SCARB2

## Abstract

Enterovirus 71 (EV-A71) is one of the major causative agents of hand, foot, and mouth disease. EV-A71 infection is sometimes associated with severe neurological diseases such as acute encephalitis, acute flaccid paralysis, and cardiopulmonary failure. Therefore, EV-A71 is a serious public health concern. Scavenger receptor class B, member 2 (SCARB2) is a type III transmembrane protein that belongs to the CD36 family and is a major receptor for EV-A71. SCARB2 supports attachment and internalization of the virus and initiates conformational changes that lead to uncoating of viral RNA in the cytoplasm. The three-dimensional structure of the virus-receptor complex was elucidated by cryo-electron microscopy. Two α-helices in the head domain of SCARB2 bind to the G-H loop of VP1 and the E-F loop of VP2 capsid proteins of EV-A71. Uncoating takes place in a SCARB2- and low pH-dependent manner. In addition to SCARB2, other molecules support cell surface binding of EV-A71. Heparan sulfate proteoglycans, P-selectin glycoprotein ligand-1, sialylated glycan, annexin II, vimentin, fibronectin, and prohibitin enhance viral infection by retaining the virus on the cell surface. These molecules are known as “attachment receptors” because they cannot initiate uncoating. In vivo, SCARB2 expression was observed in EV-A71 antigen-positive neurons and epithelial cells in the crypts of the palatine tonsils in patients that died of EV-A71 infection. Adult mice are not susceptible to infection by EV-A71, but transgenic mice that express human SCARB2 become susceptible to EV-A71 infection and develop neurological diseases similar to those observed in humans. Attachment receptors may also be involved in EV-A71 infection in vivo. Although heparan sulfate proteoglycans are expressed by many cultured cell lines and enhance infection by a subset of EV-A71 strains, they are not expressed by cells that express SCARB2 at high levels in vivo. Thus, heparan sulfate-positive cells merely adsorb the virus and do not contribute to replication or dissemination of the virus in vivo. In addition to these attachment receptors, cyclophilin A and human tryptophanyl aminoacyl-tRNA synthetase act as an uncoating regulator and an entry mediator that can confer susceptibility to non-susceptibile cells in the absence of SCARB2, respectively. The roles of attachment receptors and other molecules in EV-A71 pathogenesis remain to be elucidated.

## Background

**Fig. 1:**
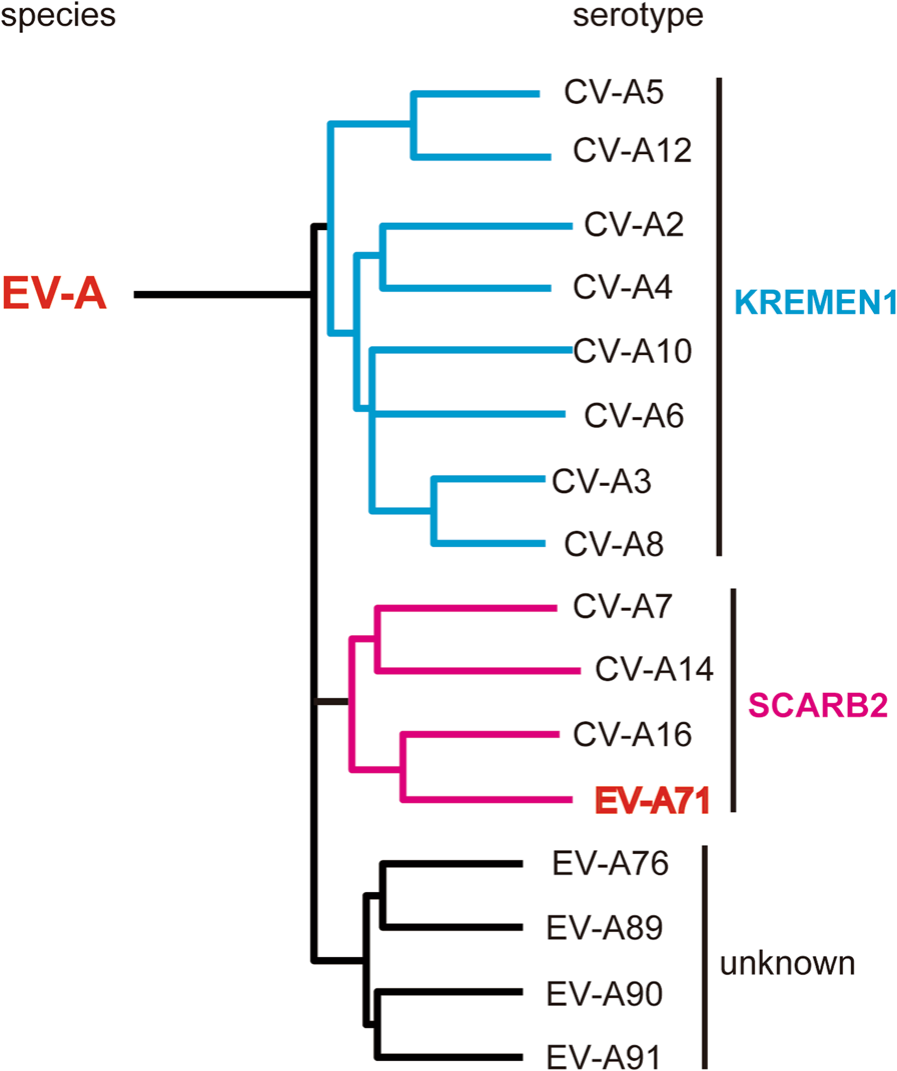
EV-A and receptor usage. There are 25 serotypes in EV-A. Sixteen serotypes whose natural host is human are shown. A group of closely related viruses (EV-A71, CV-A16, CV-A14 and CV-A7), use SCARB2 as the main receptor. EV-A71 also uses attachment receptors. Other groups, including CV-A2, CV-A3, CV-A4, CV-A5, CV-A6, CV-A8, CV-A10, and CV-A12, use KREMEN1

Human enteroviruses (HEVs) belonging to the genus *Enterovirus* within the family *Picornaviridae* are non-enveloped viruses with a single-stranded RNA genome of positive polarity. EVs comprise 15 species (EV-A to L and Rhinovirus-A to C). EV-A includes at least 16 members with different serotypes–Coxsackievirus (CV)-A2, CV-A3, CV-A4, CV-A5, CV-A6, CV-A7, CV-A8, CV-A10, CV-A12, CV-A14, CV-A16, enterovirus A71 (EV-A71), EV-A76, EV-A89, EV-A90, and EV-A91, which were formerly named human enterovirus A (Fig. 1) [1]. EV-As cause hand, foot, and mouth disease (HFMD), herpangina, meningitis, polio-like flaccid paralysis, and respiratory disease [2, 3]. EV-A71 and CV-A16 are the major causative agents of HFMD. In addition to these viruses, outbreaks of HFMD caused by CV-A6 have been increasing since 2008 [4]. HFMD is normally a mild disease in which patients develop vesicular lesions on the hands, foot and mouth; however, HFMD caused by EV-A71 is sometimes associated with severe neurological complications such as acute fatal encephalitis, polio-like acute flaccid paralysis, and neurogenic pulmonary edema. Recently, repeated outbreaks of EV-A71 with severe neurological complications have occurred in the Asia-Pacific region [5–18] and have become a serious public health concern. In this review, we summarize recent studies on EV-A71 receptors and discuss the roles of these molecules in the pathogenicity of EV-A71.

Viral receptors can be a primary determinant of species-specific and tissue-specific infection because enterovirus receptors mediate the initial steps of virus infection, including binding to the cell surface, internalization, and initiation of conformational changes in the virion that lead to uncoating [19]. Therefore, it is important to elucidate the molecular mechanisms underlying these early steps of infection in order to understand the pathogenicity of the virus and to develop strategies to prevent viral diseases.

Humans are the natural host of EV-As. Old-world primates such as cynomolgus monkeys and rhesus monkeys are not natural hosts, but they are susceptible to EV-A infection and can be infected with EV-As experimentally [20–23]. Neonatal mice can also be experimentally infected with EV-As; this can be achieved by inoculating them (via the intracerebral, intraperitoneal, and subcutaneous routes) with virus isolated from swabs taken from HFMD patients. The virulence of the virus can be evaluated using neonatal mouse model [24–28]. Efficient viral replication occurs in the central nervous system (CNS) and muscle of infected mice. Neonatal mice are susceptible to EV-A infection for less than 2 weeks. Thus, it seems that the EV-As receptors in humans and other primates are different from those in neonatal mice. The receptors for human infection have been studied extensively, while those for infection of neonatal mice have not.

The capsid structures of closely related EV-As are similar, and they therefore utilize the same receptors for infection. EV-As are now classified into at least two major groups according to the receptor used when infecting human cells (Fig. 1) [29, 30]. One group consists of EV-A71, CV-A7, CV-A14 and CV-A16, which are members of one monophyletic group. These viruses use human scavenger receptor class B, member 2 (hSCARB2) as the major receptor [29, 31]. Recently, KREMEN1 was identified as a receptor for the prototype strain of CV-A10 [30]. KREMEN1 is also used as a receptor by another group of EV-As, CV-A2, CV-A3, CV-A4, CV-A5, CV-A6, CV-A8, CV-A10, and CV-A12, which are in another monophyletic group. Receptors for the remaining EV-As (EV-A76, EV-A89, EV-A90, and EV-A91) have not been identified.

**Fig. 2:**
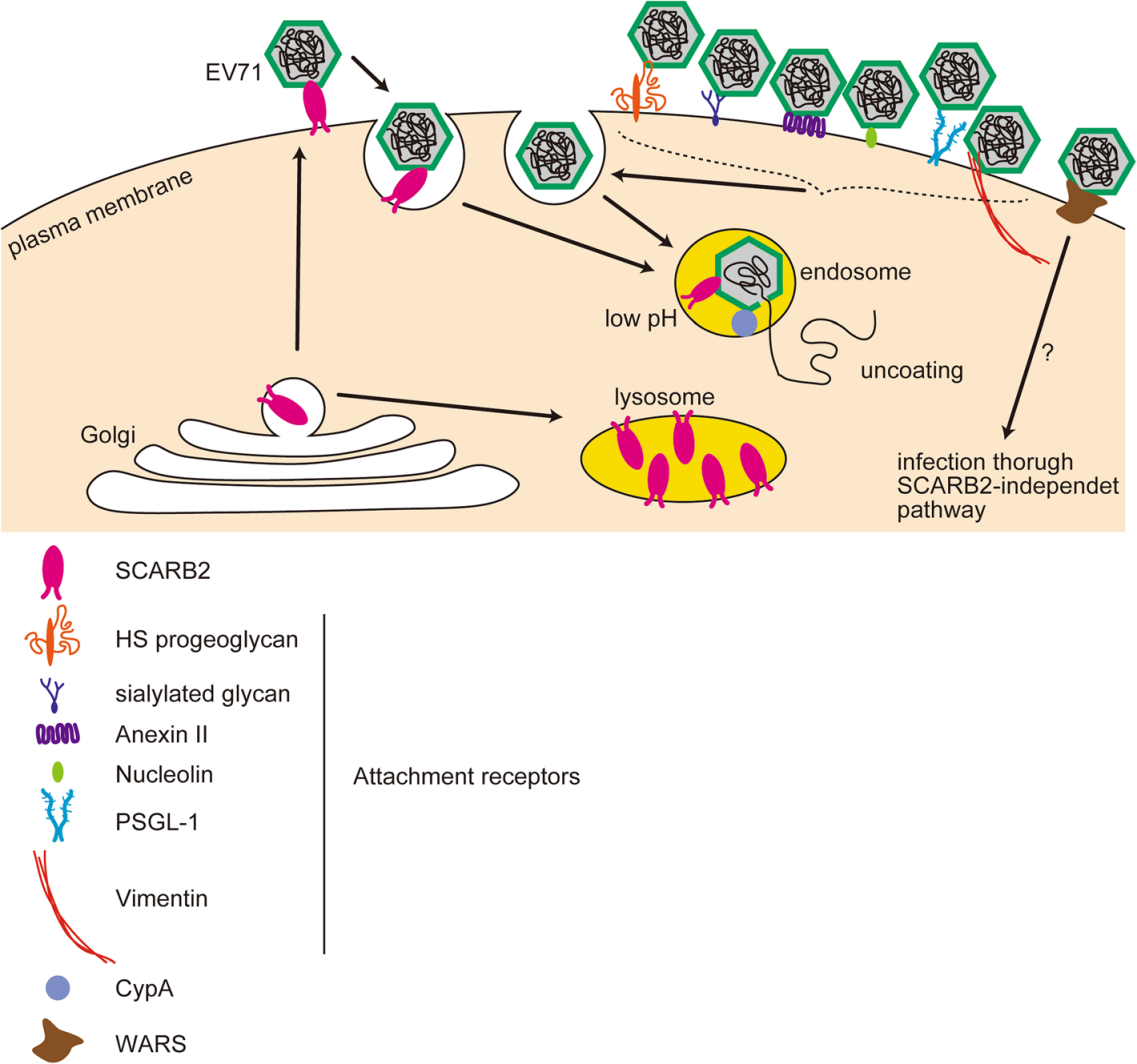
Role of SCARB2 and attachment receptors. SCARB2 is expressed abundantly in lysosomes but not at the cell surface. EV-A71 enters cells using attachment receptors. Attachment receptors cannot initiate conformational changes in the virion. Internalized viruses may encounter SCARB2 in endosomes, where uncoating takes place after acidification of these endosomes. hWARS may mediated a infection pathway distict from the SCARB2-dependent pathway. CypA may be involved in uncoating

The best-characterized enterovirus receptor is the poliovirus receptor (PVR, CD155) [32, 33]. Studies on PVR are important in that they facilitate comparative understanding of other enterovirus receptors. The PVR alone is sufficient to mediate cell surface binding, internalization, and initiation of conformational changes of the virion that lead to uncoating. The species specificity of poliovirus is determined by expression of its cognate receptor. Expression of the PVR is sufficient to make non-susceptible mouse cells susceptible to poliovirus. Mice become susceptible to poliovirus infection after transgenic (tg) expression of the human PVR [34, 35]. PVR tg mice develop neurological diseases similar to those in infected humans and monkeys. In both humans and PVR tg mice, the PVR is expressed in a wide variety of tissues, including the CNS (in which poliovirus replicates most efficiently) and other tissues that are not targets of poliovirus replication. Therefore, the PVR is required to establish in vivo infection, although its expression does not in itself determine whether specific cell types are susceptible to poliovirus infection; other factors such as innate immune responses play a role [36, 37]. However, EV-A71 infection is not as simple as poliovirus infection. During EV-A71 infection, hSCARB2 plays pivotal roles in attachment, internalization, and uncoating, but it is not the only receptor that supports infection. In studies using cultured cells, it has been shown that other molecules such as P-selectin glycoprotein ligand-1 (PSGL-1) [38], annexin II (Anx2) [39], vimentin [40], nucleolin [41], heparan sulfate (HS) proteoglycan [42], sialylated glycan [43], fibronectin [44], and prohibitin [45] support viral attachment to the cell surface but cannot induce conformational changes in the virion that lead to uncoating; therefore, they are called “attachment receptors” (Fig. 2). In addition, molecules that are not defined as attachment receptors are involved. For example, cyclophilin A (CypA) enhances uncoating of the virion [46], and human tryptophanyl aminoacyl-tRNA synthetase (hWARS) makes non-susceptible cells susceptible in the absence of SCARB2 [47]. The pathogenicity of EV-A71 may depend on these molecules.

## EV71 receptors

### SCARB2

Human RD cells and monkey Vero cells, but not mouse L929 cells (which lack appropriate receptors), are susceptible to infection by EV-A71. Yamayoshi et al. [31] found that transfection of mouse L929 cells with human *SCARB2* gene conferred susceptibility infection. SCARB2, also known as lysosomal integral membrane protein II (LIMP-II), LGP85, and CD36b like-2, belongs to the CD36 family [48, 49]. It is a type III double-transmembrane protein of 478 amino acids, with a large exofacial domain and short cytoplasmic domains at the amino- and carboxyl-termini [48]. Physiologically, SCARB2 is involved in membrane transport and reorganization of the endosomal/lysosomal compartment [49–51]. SCARB2 mediates delivery of β-glucocerebrosidase (β-GC) from the endoplasmic reticulum to lysosomes [52]. Thus, SCARB2 is localized predominantly to the lysosomal membrane; only a small proportion is present in the plasma membrane (Fig. 2).

The crystal structure of the SCARB2 ectodomain has been elucidated [53, 54]. SCARB2 comprises a large anti-parallel β-barrel with many short α-helical segments. Two α-helices, α1 and α15, are connected to the amino-terminal and carboxyl-terminal transmembrane regions at the bottom, respectively. The head region at the top of the β-barrel fold comprises a three α-helix bundle consisting of α4, α5, and α7, two other short helices (α2 and α14), and the β7 strand. The three-dimensional structure of SCARB2 changes depending on the environmental pH [54]. A histidine residue at position 150 of hSCARB2 is a key amino acid for switching between the neutral form, which binds β-GC, and the acidic form, which does not [55]. Nine *N*-glycosylation sites are present in SCARB2, but the head region is free of carbohydrate chains.

**Fig. 3:**
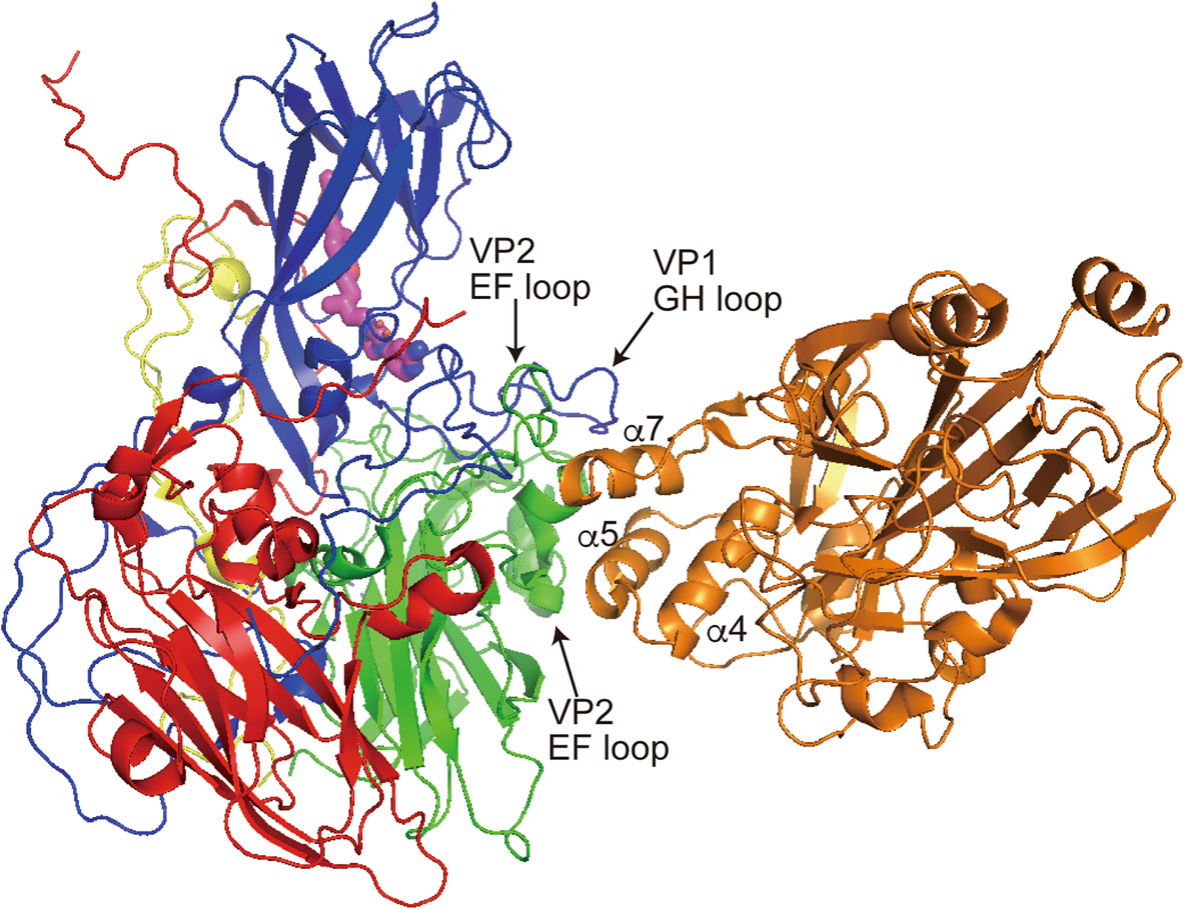
Three-dimensional structure of the EV-A71-SCARB2 complex. The 3D structure of EV-A71 capsid protomer (VP1, VP2, VP3, and VP4 in blue, green, red, and yellow, respectively) and ectodomain of SCARB2 (orange) are shown. α5 and α7-helices of SCARB2 contact with the G-H loop of VP1 and E-F loops of VP2, which form southern rim of the canyon. The cavity for pocket factor (magenta) is distant from the SCARB2 binding site. Carbohydrate chains are not indicated. This figure is produced from Protein Data Base 6I2K

SCARB2 can bind EV-A71 virions directly, as demonstrated biochemically in pull-down assays [31]. Binding of EV-A71 to the cell surface is increased by expression of hSCARB2. The region of SCARB2 required for EV-A71 binding and infection was identified using chimeric mutants of human and mouse SCARB2 [56]. Chimeras that contained amino acids 142–204 of the human sequence, which are encoded by exon 4 of the *SCARB2* gene, are functional receptors for EV-A71. This region of the SCARB2 protein corresponds to the head region and determines species-specific infection of cultured cells by EV-A71. Enzymatic removal of the carbohydrate moiety from the recombinant soluble SCARB2 protein did not abolish virus binding to the receptor. Recently, the EV-A71-SCARB2 complex structure was determined at 3.4 Å resolution using cryo-electron microscopy [57]. This analysis revealed that α5(153–163) and α7 (183–193) of SCARB2 are the main sites of contact with the virion (Fig. 3).

Infection by EV-A71 requires acidification of endosomes. Therefore, uncoating is thought to occur in a SCARB2-dependent and low pH-dependent manner. Yamayoshi et al. [58] demonstrated that incubation of EV-A71 with soluble SCARB2 induced a conformational change at an acidic pH (below 6.0). After this treatment, an empty capsid composed of VP1, VP2, and VP3 (without genomic RNA) was detected by sucrose density gradient centrifugation. Other uncoating receptors, such as ICAM-1 for major group human rhinoviruses, PVR for poliovirus, and Coxsackie-adenovirus receptor (CAR) for coxsackievirus B, bind inside of the canyon and expel the pocket factor away from the cavity at the floor of the canyon [59–63]. However, study of the EV-A71-SCARB2 complex revealed that SCARB2 does not bind inside the canyon but rather at its southern rim, such that the VP1 G-H loop and the VP2 E-F loops are the main contact sites on EV-A71 [57] (Fig. 3). The authors hypothesized that the pH-dependent conformational change within SCARB2 distorts the VP1 G-H loop so that the pocket factor is expelled via an allosteric effect.

SCARB2 is not expressed ubiquitously, although it is expressed in a variety of human tissues [64, 65]. High expression of SCARB2 is observed in neurons within the CNS, and in lung pneumocytes, hepatocytes, splenic germinal centers, renal tubular epithelium, and intestinal epithelium. In fatal human cases, EV-A71 antigens were detected in CNS neurons and in epithelial cells lining the crypts of the palatine tonsils; both are areas where SCARB2 is expressed [65]. Therefore, SCARB2 is thought to play an essential role in infection in vivo. Other evidence was obtained using tg mouse experiments. EV-A71 infects neonatal mice but cannot infect or cause disease in adult mice. Fujii et al. [64] produced tg mice that express human SCARB2 driven by its own promoter. The expression profile of human SCARB2 in these mice was similar to that in humans. When tg mice, up to 21 weeks old, were inoculated with EV-A71 via the intracerebral, intravenous, or intraperitoneal routes, they exhibited paralytic disease similar to that observed in fatal human cases. EV-A71 antigens were detected in neurons in the brainstem, the cerebellar nuclei, and spinal cord of SCARB2 tg mice. Yang et al. [66] recently produced similar tg mice expressing hSCARB2 driven by the mouse *Scarb2* promotor. These results suggest that expression of SCARB2 alone is sufficient to cause neurological disease in mice. Lin et al. [67] generated another tg mouse model that expressed human SCARB2 using a ubiquitous promoter. However, tg mice older than 3 weeks were not susceptible to EV-A71, and the main EV-A71 replication site in the neonatal tg mice (unlike humans) was skeletal muscle. Zhou et al. [68] generated SCARB2 knock-in mice in which SCARB2 cDNA driven by the CAG promoter was inserted into the ROSA26 locus. These knock-in mice were susceptible to EV-A71 infection. However, susceptibility to EV-A71 was decreased after the age of 3 weeks [69], similar to that in mice established by Lin et al. [67]. Thus, two mouse models that express SCARB2 via a ubiquitous promotor are less vulnerable to EV-A71 infection. These results suggest that expression of SCARB2 at appropriate sites is important for mimicking pathogenicity in humans. A similar phenomenon was observed in PVR tg mice [70]. PVR tg mice in which the PVR was expressed under control of the human PVR promoter showed PV infection of neurons, with a fatal outcome. Other PVR tg mice in which the PVR was expressed under the control of a ubiquitous CAG promoter were also susceptible to PV; however, a fatal outcome was observed only when mice received an extremely high dose of PV [70].

### HS

HS is a linear polysaccharide comprising repeating disaccharide units of N-acetylated or N-sulfated glucosamine and glucuronic acid or iduronic acid [71], which are highly negatively charged due to their sulfate groups. HS proteoglycans comprise core proteins, mainly syndecans and glypicans, with covalently attached HS chains [72]. The HS chains serve as ligands for a large number of proteins, including many viruses [73–81]. Tan et al. [42] provided several lines of evidence that HS acts as an surface attachment receptor for a subset of EV-A71 on RD cells. EV-A71 particles bind to heparin-Sepharose columns at physiological salt concentrations. Preincubation of EV-A71 with HS analogs such as heparin, polysulfated dextran sulfate, or suramin inhibit EV-A71 infection of RD cells. In addition, EV-A71 infection or cell surface binding is reduced when HS biosynthesis is blocked with sodium chlorate, by knockdown of N-deacetylases/N-sulfotransferase-1 and exostosin-1, or when HS is removed by heparinase I/II/III treatment.

Tan et al. [82] found that the lysine residues at 162, 242, and 244 of the VP1 capsid protein are responsible for electrostatic interactions with HS. When mutations were introduced at these residues, cell binding was reduced significantly, although the HS-nonbinding mutants acquired compensatory mutations rapidly. Mutations of VP1 at other residues influence HS-binding ability. For example, a double mutant (VP1-98E and -145E) does not bind HS at all, although it acquired compensatory mutations (VP1-98 K or -145Q/G) rapidly, which restored HS-binding. These results suggest that multiple positively charged residues close to the five-fold axis determine HS adaptation. Consistent with this, passage of EV-A71 in cell culture often induces mutations in capsid proteins [83]. These results suggest that conversion from HS-nonbinding strains to HS-binding mutants is associated with adaptation of the virus to cell culture, and that this occurs very frequently due to the abundant expression of HS on the surface of cultured cells. This points to the advantage of using HS as the attachment receptor and suggests that this is the mechanism that drives emergence of HS-binding strains in cell culture.

The role of HS in viral dissemination and pathogenesis in vivo has been investigated using hSCARB2 tg mice and cynomolgus monkey models. Kobayashi et al. [84] compared the pathogenicity of HS-binding and -nonbinding mutants (VP1–145G and VP1–145E, respectively) after inoculation into hSCARB2 tg mice intravenously. The HS-nonbinding mutant (VP1–145E) was more virulent than the HS-binding mutant (VP1–145G). Immunohistochemical staining revealed that HS is expressed at high levels by vascular endothelial cells and some other cell types such as sinusoidal endothelial cells in the liver and the glomerulus of the kidney, areas in which hSCARB2 is expressed at low or undetectable levels. This result suggests that HS-binding strains bind to some cells in which the virus cannot replicate in the absence of SCARB2. By contrast, CNS neurons (where the virus replicates efficiently) express high levels of hSCARB2 but low levels of HS. Consequently, the VP1–145G virus was undetectable in the bloodstream shortly after inoculation into hSCARB2 mice. This trapping effect was not observed when mice were inoculated with VP1–145E. These data suggest that the VP1–145G virus is adsorbed by the attachment receptor (HS) in vivo, leading to abortive infection of HS-positive cells. This effect is thought to be a major mechanism by which the VP1–145G virus is attenuated. Thus, the HS attachment receptor inhibits rather than increases dissemination of HS-binding viruses. Similar results were obtained by Fujii et al. [85] using cynomolgus monkeys. More recently, Tee et al. [86] generated a number of mutants that showed different degrees of heparin binding activity. They showed that weak heparin binders have a more virulent phenotype than strong heparin binders in a neonatal mouse model. The weak heparin-binders inoculated into mice disseminated efficiently and displayed high viremia. The initially strong heparin-binding variant acquired an additional mutataion, which confers weak heparin-binding phenotype and high virulence. Furthermore, attenuation of viruses via cell culture adaptation mediated by glycosaminoglycans (including HS) has been reported for many *Flaviviridae* (e.g., Japanese encephalitis virus, Murray Valley encephalitis virus, West Nile virus, and Dengue virus) [87–90], *Togaviridae* (Sindbis virus, Venezuelan equine encephalitis virus, Tick-borne encephalitis virus, and Chikungunya virus) [91–94], and *Picornaviridae* (human Rhinovirus (HRV) C15, HRV89, and foot and mouth disease virus) [95–97]. In addition to this trapping effect, Fujii et al. [85] reported that HS-binding strains are more easily neutralized by antibodies than HS-nonbinding strains. Thus, HS-binding EV-A71 strains are less able to disseminate throughout the body of an animal for at least two reasons: they are trapped by HS, and they are easily neutralized by antibodies. Nishimura et al. [98] analyzed the abundance of mutants using all sequence data available in GenBank and found that approximately 80% of EV-A71 strains were of the HS-nonbinding type. In this analysis, they simply counted the number of viruses with an HS-binding or -nonbinding phenotype in the database without knowing anything about the passage history in cultured cells and/or the condition of the patients from which they were isolated. Considering that the mutations occur during propagation of isolated viruses in cell culture, the abundance of HS-binding types may be much lower than thought. Indeed, Mizuta et al. [99, 100] determined the VP1 sequence of a large number of EV-A71 strains freshly isolated from HFMD patients and submitted them to GenBank. All clinical isolates of EV-A71 had an E residue at VP1–145. These results suggest that the HS-nonbinding strains are dominant in humans.

By contrast, other studies reported isolation of a HS-binding strain from an immunocompromized patient [101, 102]. The HS-binder was not detected in the respiratory tract, but it was detected in the blood, cerebrospinal fluid, and stool. The authors thought that the HS-binding mutants arose “in host” and disseminated to those tissues. They also showed that the HS-binding phenotype contributed to positive selection in tissue culture models that mimicked upper and lower respiratory airway epithelia and intestinal and neural tissues. They claimed that the HS attachment receptor played a critical role in EV-A71 virulence, and that “in host” EV-A71 adaptation to a HS-dependent virus was likely responsible for its dissemination. Thus, under specific conditions, HS-binding strains might have an advantage with respect to disseminating throughout the body.

### PSGL-1

PSGL-1 is a glycoprotein that functions as a high affinity counter-receptor for the cell adhesion molecules P-, E- and L-selectin [103–105]. This protein plays an important role in leukocyte trafficking during inflammation by tethering leukocytes to activated platelets or endothelial cells expressing selectins. PSGL-1 is expressed by lymph node dendritic cells and macrophages in the intestinal mucosa [103]. Nishimura et al. [38] used a panning procedure to show that PSGL-1 binds to the EV-A71 1095 strain. This method is suitable for screening molecules that have a high affinity for EV-A71 virions, but it is not an assay that can confirm establishment of infection. Initially, it was reported that PSGL-1 made non-susceptible cells susceptible to EV-A71. The PSGL-1-EV71 complex is able to enter the cell via a caveolin-dependent pathway, and disturbing caveolar endocytosis using specific inhibitors (genistein and flipin) or the use of caveolin-1 siRNA in Jurkat and L-PSGL-1 cells significantly inhibits EV71 infection [106]. However, EV-A71 does not infect PSGL-1-expressing cells efficiently unless used at an extremely high multiplicity of infection and the cells are exposed to the virus for long time. Later, the same authors reported that EV-A71 did not infect L929 cells expressing PSGL-1 (L-PSGL-1) efficiently, and that mutations in the capsid protein VP2 were required for efficienty infectivity [107]. Yeung et al. [47] could not confirm efficient infection in L-PSGL-1 cells. Indeed, PSGL-1 shows no uncoating activity [58]. Infection of L-PSGL-1 cells might be achieved by uncoating events mediated via thermal destabilization of a virion that has been captured by PSGL-1 for a long time. Thus, PSGL-1 may be classified as an attachment receptor. Human PSGL-1 binds EV-A71 via three sulfated tyrosine residues at positions 46, 48, and 51 close to the amino-terminus of PSGL-1 [108]. It should be noted that not all EV-A71 viruses bind PSGL-1. Thus, EV-A71 can be divided into two groups: PSGL-1-binding strains (PB) and PSGL-1 nonbinding strains (non-PB). PSGL-1 binds to positively charged amino acids located near the five-fold axis of the EV-A71 virion via an electrostatic interaction [98]. VP1–145 of EV-A71 affects the surface structure of the virion and determines the PSGL-1-binding phenotype. Lysine residues located at positions 242 and 244 of VP1 are highly exposed on the surface in PB strain virions (VP1–145G/Q). By contrast, these amino acids are less exposed in the non-PB (VP1–145E) strains. According to available sequence data, approximately 80% of EV-A71 isolates are non-PB.

The role of PSGL-1 during in vivo infection is not clear. In fatal human cases, EV-A71 antigens were detected in crypt epithelial cells in the palatine tonsils and in neurons of the CNS [65]. However, no expression of PSGL-1 was observed in these cells [65], suggesting that PSGL-1 is not involved in infection. In addition, tg expression of PSGL-1 in mice did not confer susceptibility [109]. Kataoka et al. [110] examined whether PB strains are able to infect cynomolgus monkeys more efficiently. EV-A71-PB was undetectable in the bloodstream shortly after inoculation and did not show high virulence, while EV-A71-non-PB was more pathogenic. Binding of EV-A71 to PSGL-1 is mediated by an electrostatic interaction [98]. Therefore, the binding specificity of EV-A71 for PSGL-1 resembles that of EV-A71 for HS. In support of this idea, a suramin derivative (NF449) inhibits interaction of the virus with PSGL-1 and HS [111]. PB strains may be captured by HS, resulting in an attenuated phenotype.

Recently, Sun et al. [112] showed that a mouse-adapted EV-A71 strain increased virulence by acquiring an additional mutation in the VP2 capsid protein, thereby allowing binding to mouse PSGL-1. However, mouse PSGL-1 does not usually bind wild-type EV-A71. Therefore, it is unclear whether these data support the notion that human PSGL-1 plays a role in human infection.

### Anx2

Anx2 is a multifunctional protein involved in endocytosis, exocytosis, membrane domain organization, actin remodeling, signal transduction, protein assembly, transcription and mRNA transport, and DNA replication and repair [113]. Anx2 is expressed in the majority of cells and tissues and binds to numerous ligands. Yang et al. [39] used a virus overlay-protein binding assay to detect a 36 KDa protein in RD cell lysates that binds to EV-A71 virions. This protein was identified as Anx2 by mass spectrometry. Direct binding of five different genotypes of EV-A71 to Anx2 was demonstrated using pull-down assays. Anx2 did not bind CV-A16 in that assay, suggesting that binding was specific for EV-A71. Pretreatment of EV-A71 with soluble recombinant Anx2, or pretreatment of host cells with an anti-Anx2 antibody, reduced viral attachment to the cell surface and the virus yield. HepG2 cells that stably expressed Anx2 generated significantly higher viral titers than parental HepG2 cells, suggesting that Anx2 increased infection. Using yeast two-hybrid analysis, the Anx2-interacting domain on the VP1 capsid protein was mapped to amino acids 40–100, which comprise β-sheet B and part of the B-C loop. Viral entry and uncoating via Anx2 have not been reported.

### Sialylated glycans

Sialic acid is present on terminal monosaccharides expressed on the glycan chains of glycolipids and glycoproteins [114], which are distributed widely throughout almost all tissues and used as receptor by many viruses. DLD-1 intestinal cells are susceptible to infection by EV-A71; Yang et al. [43] hypothesized that sialylated glycans on DLD-1 cells might be recognized as EV-A71 receptors. Depletion of *O*-linked glycans using the *O*-linked glycan synthesis inhibitor benzyl *N*-acetyl α-*D*-galactosamine inhibits EV-A71 infection. Pretreatment with α2,3 and α2,6 sialidase reduces EV-A71 replication in DLD-1 cells significantly. Furthermore, addition of sialic acid-α2,3-linked galactose and sialic acid-α2,6-linked galactose (purified from human milk) to cell cultures inhibits EV-A71 infection of DLD-1 cells significantly. These results suggest that sialic acid-linked glycans are responsible for EV-A71 infection of DLD-1 cells. However, no direct interaction between sialylated glycans and EV-A71 has been proved.

### Nucleolin

Nucleolin is a multifunctional eukaryotic nucleolar phosphoprotein [115] located mainly in dense fibrillar regions of the nucleolus. It is also expressed at the cell surface where it acts as a receptor for human immunodeficiency virus (HIV) [116] and respiratory syncytial virus (RSV) [117]. Su et al. [41] performed a glycoproteomics analysis of membrane proteins expressed by RD cells. They purified sialylated glycoproteins from cell membrane extracts using lectin chromatography and treated them with sialidase, followed by immunoprecipitation with EV-A71 particles. One candidate EV-A71 binding partner was nucleolin. ELISA suggested that EV-A71 interacted with nucleolin directly via the VP1 capsid protein; in addition, an anti-nucleolin antibody inhibited binding of EV-A71 to RD cells. Knockdown of nucleolin in RD cells reduced EV-A71 binding and infection. Expression of human nucleolin in mouse NIH3T3 cells increased binding of EV-A71 and the numbers of cells showing cytopathic effects (CPE). These results suggest that nucleolin is an attachment receptor for EV-A71. However, no study has described virus internalization and uncoating after binding to nucleolin.

### Vimentin

Vimentin a type III intermediate filament protein. Intermediate filaments, along with microtubules and actin microfilaments, make up the cytoskeleton [118]. Vimentin is responsible for maintaining cell shape and the integrity of the cytoplasm, and for stabilizing cytoskeletal interactions. It is also expressed on the cell surface; indeed, cell surface vimentin plays a role in the attachment of a number of pathogens [119–123]. Du et al. [40] demonstrated that it also acts as an attachment receptor for EV-A71 using U251, RD, HeLa, and Vero cells. Direct binding of vimentin to VP1 of EV-A71 was proved by pull-down experiments. Binding of the virus to the cell surface was reduced by competition with soluble vimentin, by an anti-vimentin antibody, and by knockdown of vimentin expression using RNA interference (RNAi). The anti-vimentin antibody alone was not sufficient to block EV-A71 infection completely. The anti-vimentin antibody and an anti-SCARB2 antibody had an additive effect on inhibition of EV-A71 infection. The EV-A71 binding site in vimentin was localized to amino acids 1–57 of VP1 in in vitro assay. However, this region is localized inside the native virion. It is not clear how vimentin binds the native virion. Mouse vimentin was able to bind EV-A71, but vimentin did not bind CV-A16. These data suggest that cell surface vimentin promotes EV-A71 infection in cultured cells by acting as an attachment receptor. However, it has not been shown whether vimentin also plays a role in EV-A71 infection in vivo.

### Fibronectin

Fibronectin is a high molecular weight glycoprotein that plays important roles in cell adhesion, growth, migration, and differentiation [124]. He et al. [44] found that overexpression of fibronectin enhanced EV-A71 infection, and that knockout of fibronectin reduced viral binding to host cells and decreased viral yield. A short peptide containing an Arg-Gly-Asp (RGD) motif, which is known to inhibit interaction between integrin and fibronectin, inhibited EV-A71 infection in cultured cells and in neonatal mice. The amino-terminal half of VP1 of EV-A71 co-precipitated with the D2 domain of fibronectin, suggesting that EV-A71 and fibronectin interact through these domains. These results suggested that cellular fibronectin is an attachment receptor for EV-A71.

### Prohibitin

Prohibitin is expressed ubiquitously in multiple cellular compartments, including the mitochondria, nucleus, and plasma membrane. Mitochondrial and nuclear prohibitin have multiple functions, including cellular differentiation, anti-proliferation, and morphogenesis [125]. Too et al. [45] found that prohibitin plays a role in EV-A71 entry and intracellular replication in NSC-34 cells; these cells are a fusion between murine neuroblastoma and spinal cord cells and possess motor neuron-like properties [126]. Using a two-dimensional proteomic approach combined with mass spectrometry, the authors identified several host proteins that are upregulated in EV-A71-infected NSC-34 cells. Silencing prohibitin using siRNA led to significantly lower virus titers. Treatment with an antibody specific for prohibitin inhibited infection of NSC-34 cells by EV-A71. Co-immunoprecipitation experiments confirmed direct interaction between EV-A71 and prohibitin. A proximity ligation assay revealed that EV-A71 binds to prohibitin but not to murine Scarb2 on the surface of NSC-34 cells, suggesting that prohibitin may mediate Scarb2-independent entry. However, this result is obtained using a mouse cell line. The importance of prohibitin during EV-A71 infection of human cells remains unclear.

### Cyp A

Cyclophilins are involved in transcriptional regulation, immune responses, protein secretion, and mitochondrial function [127]. CypA has peptidyl-prolyl *cis*-*trans* isomerase activity and plays critical roles in proliferation of a number of viruses [128], Qing et al. [46] found that a CypA inhibitor also inhibits EV-A71 replication, as did knockdown of CypA. CypA binds to the H-I loop of the VP1 capsid protein. This region contains a proline residue at VP1–246. Incubation of CypA with EV-A71 virions at pH 6.0 (but not 5.5 or 6.5) alters the sedimentation coefficient of EV-A71 virions from 160 S to other forms was observed, suggesting that CypA is an uncoating regulator in a pH-dependent manner. These results suggest that CypA is a host factor that regulates uncoating, making it different from other attachment receptors reported previously.

### hWARS

Yeung et al. [47] used genome-wide RNAi library screen to identify a new entry factor for EV-A71. RD cells were transduced with a lentiviral shRNA library and cells that became resistant to EV-A71 infection were selected. Human tryptophanyl aminoacyl-tRNA synthetase (hWARS) was identified as a protein that was knocked down in EV-A71-resistant cells. hWARS catalyzes aminoacylation of tRNA (Trp) with tryptophan and is interferon (IFN)-γ-inducible [129]. Knockdown of hWARS protects RD cells from EV-A71-induced CPE, and viral replication is much lower than in control wild-type RD cells. Interestingly, inhibited viral replication was also observed when hWARS-knockout cells were infected with other EV serotypes, including CV-A16, CV-A6, echovirus 11 (E-11), E-6, E-25, E-30, and EV-D68, suggesting that hWARS plays an important role in infection by a broad spectrum of enterovirus serotypes.

EV-A71 colocalized with hWARS at the cell surface. Pull-down experiments revealed direct binding between hWARS and EV-A71. Infection with EV-A71 was inhibited by preincubation of soluble recombinant hWARS with an anti-hWARS antibody. Unlike other candidate receptors, hWARS alone was sufficient for EV-A71 infection in the absence of hSCARB2. Non-susceptible mouse L929 cells became susceptible to EV-A71 upon expression of hWARS. Furthermore, NT2 cells deficient in hSCARB2 expression were still susceptible to EV-A71 infection, but those deficient in hWARS were not. The results suggest that hWARS-mediated infection is a new pathway distinct from SCARB2-mediated infection. However, it is not known whether hWARS induces the conformational changes in the virion that lead to uncoating. To examine the role of hWARS in vivo, hWARS was overexpressed in 5-day-old mice using a lentiviral vector, and the mice were challenged with EV-A71. EV-A71 antigens and pathological changes were observed in the brain, muscle, heart, and lungs of the infected mice expressing retroviral hWARS. The authors claimed that a mouse homolog of WARS (mWARS) was expressed at high levels in the intestine, lungs, and liver, and that expression correlated strongly with the tissue tropism and pathogenesis of EV-A71. However, they did not demonstrate whether mWARS was functional, and they did not explain why adult mice lost susceptibility despite expressing mWARS. These issues should be examined in future studies.

## Conclusions

To date, hSCARB2 is the only receptor known to have three important functions in EV-A71 infection: virus binding, internalization, and initiation of uncoating. However, hSCARB2 is a lysosomal protein not abundantly expressed on the cell surface. Therefore, the virus must utilize other attachment receptors to achieve efficient infection. Most of these alternative attachment receptors cannot initiate uncoating. The involvement of attachment receptors is demonstrated during infection of cultured cells, and most were reported in only a single publication [39–41, 43–45]. Therefore, neither the mode of internalization nor the uncoating activity has been confirmed. Subsequent publications provide no further supporting evidence.

hWARS and CypA might belong to a different category from the above-mentioned attachment receptors. Cells expressing hWARS become susceptible to EV-A71 infection even in the absence of hSCARB2, although the uncoating activity of hWARS has not been demonstrated. CypA does play a role in uncoating. The molecular mechanisms by which these molecules act during the early events of EV-A71 infection remain unclear.

Overall, the roles of EV-A71 receptors in vivo are poorly understood. Among them, HS and PSGL-1 have been characterized in some detail. Although HS does increase viral infection of cultured cells, it (and possibly PSGL-1) actually inhibits EV-A71 infection in vivo. The significance of other attachment receptors in vivo should be determined in future studies.

## Data Availability

Not applicable.

## Abbreviations

Anx2: Annexin II
CAR: Coxsackievirus-adenovirus receptor
CV: Coxsackievirus
EV: Enterovirus
HFMD: Hand, foot, and mouth disease
HS: Heparan sulfate
ICAM-1: Intercellular adhesion molecule-1,
PSGL-1: P-selectin glycoprotein ligand-1
PVR: Poliovirus receptor
SCARB2: Scavenger receptor class B, member 2
WARS: tryptophanyl aminoacyl-tRNA synthetase
